# The probability for a Pap test to be abnormal is directly proportional to HPV viral load: results from a Swiss study comparing HPV testing and liquid-based cytology to detect cervical cancer precursors in 13 842 women

**DOI:** 10.1038/sj.bjc.6602728

**Published:** 2005-08-02

**Authors:** G Bigras, F de Marval

**Affiliations:** 1 Laboratoire Cytopath, Unilabs SA, 12 Place Cornavin, Genève 1, CH-1211, Switzerland; 2 Laboratoire Bioanalytique-Riotton, Unilabs SA, 12 Place Cornavin, Genève 1, CH-1211, Switzerland

**Keywords:** HPV, liquid-based cytology, viral load sensitivity

## Abstract

In a study involving 13 842 women and 113 gynaecologists, liquid-based cytology and HPV testing for detecting cervical cancer were compared. A total of 1334 women were found to be positive for one or both tests and were invited for colposcopy with biopsy. A total of 1031 satisfactory biopsies on 1031 women were thereafter collected using a systematic biopsy protocol, which was random in the colposcopically normal-appearing cervix or directed in the abnormal one. In all, 502 women with negative tests were also biopsied. A total of 82 histologic high-grade squamous intraepithelial lesion (HSIL) were reported in biopsies, all from the group with one or both tests positive. Sensitivity and specificity to detect histologic HSIL were 59 and 97% for cytology, and 97 and 92% for HPV. In total, 14% of reviewed negative cytological preparations associated with histologic HSIL contained no morphologically abnormal cells despite a positive HPV test. This suggested a theoretical limit for cytology sensitivity. HPV viral load analysis of the 1143 HPV-positive samples showed a direct relationship between abnormal Pap test frequency and HPV viral load. Thus, not only does the HPV testing have a greater sensitivity than cytology but the probability of the latter being positive can also be defined as a function of the associated HPV viral load.

The Pap test is currently central to the detection of cervical cancer precursors. Conventional smears, whose utilisation started in the early 1950s, have been progressively replaced by liquid-based methods over the past 10 years. Discovery of the role of HPV in the natural history of cervical cancer and the ability to detect it in liquid cytology medium are rapidly modifying the standard in cervical cancer detection. HPV detection is nowadays mainly used in atypical squamous cells of undetermined significance (ASC-US) triage. The next step will be the utilisation of combined HPV testing and cytology to detect cervical cancer and its precursors. After 50 years of cytology as the sole test in cervical cancer prevention, new methods are appearing to palliate the Achilles’ heel of cytology, which is sensitivity. Indeed, many reports (Belinson *et al*, 1999; Kuhn *et al*, 2000; Ratnam *et al*, 2000; Schiffman *et al*, 2000; Wright *et al*, 2000; Clavel *et al*, 2001; Kulasingam *et al*, 2002; Petry *et al*, 2003) have established a higher sensitivity for HPV than for cytology, although published results are not unanimous (de Cremoux *et al*, 2003). The main study objective was to confirm the better sensitivity of HPV testing, especially in the context of routine clinical practice.

Between April 2002, and January 2004, a prospective clinical study was conducted in order to compare two tests for the detection of cervical cancer precursors: a liquid-based cytology preparation, Surepath® (Tripath Imaging, Burlington, NC, USA) and a test for high-risk HPV DNA, Hybrid Capture® II (Digene Corporation, Gaithersburg, MD, USA). The investigation was conducted in the laboratory Cytopath based in Geneva, Switzerland, a private laboratory member of the Unilabs Group. Cytopath has used liquid-based cytology since 1996, analysing approximately 100 000 slides per year.

## MATERIAL AND METHODS

A target of 15 000 cytologic samples was established in the hope of collecting approximately 250 histologic HSIL, providing adequate power to estimate the differences, if any, in performance characteristics between the tests. Women 30 or more years of age had been favoured for study participation in order to optimise HSIL collection. Most study participants had been screened yearly with liquid-based cytology starting at least 5 years before this study. They were thereby considered to be at low risk for cervical cancer. Recruitment was not from sexually transmitted disease clinics or other high-risk populations. Study participants were recruited by 113 gynaecologists from six Swiss cantons (Genève, Vaud, Neuchâtel, Fribourg, Valais and Tessin), most of whom are in private practice. Participants were given the option of study participation following a discussion with their gynaecologists of the risks and benefits of enrolment, and the completion of the informed consent document. Prior to initiation, the study was reviewed and approved by the Ethics Committee of the ‘Association des Médecins du canton de Genève’.

### Liquid-based cytology

All cervical samples were taken using the Surepath® (Tripath Imaging, Burlington, NC, USA) liquid-based preparation. In agreement with the manufacturer, the gynaecologists put the collecting device in the Surepath vial after having performed a cervical scrape. Cervex Brush® (CooperSurgical Inc., Trumbull, CT, USA) was the only provided collecting device. All vials were sent to the Cytopath Laboratory and prepared according to the protocol recommended by the manufacturer. A homogenised fraction of the sample was used to prepare the cytological preparation with the AutocytePrep® processor. The residual cellular material underwent centrifugation and was resuspended in 500 *μ*l distilled water for further HPV testing. In the text, ‘CYTO’ refers to the Surepath® liquid-based cytology test.

### HPV testing

HPV testing was performed at the Bioanalytique-Riotton Laboratory (another member of the Unilabs group). Cell suspensions were stored at 2–8°C for up to 1 week before HPV testing. The HPV DNA assay was performed blind to cytologic and histologic diagnoses. Specimens were tested for high- and intermediate-risk HPV types (16, 18, 31, 33, 35, 39, 45, 51, 52, 56, 58, 59 and 68) using the Digene High-Risk HPV DNA Test Hybrid Capture® II (Digene Corporation, Gaithersburg, MD, USA). The DNA assay was processed according to the manufacturer's instructions, using microtubes and a water bath for hybridisation and manual washing. In agreement with the kit manufacturer, clinical specimens were denatured for 90 min in order to reduce borderline results. Relative light units (RLU) were measured on a DML 2000 luminometer (Digene Corporation, Gaithersburg, MD, USA). Quality control was assessed using the manufacturer's assay calibration criteria and by the regular use of HC2 HPV DNA Test Panel (Digene Corporation, Gaithersburg, MD, USA). The cutoff value was calculated as the mean RLU value of the calibrator (1.0 pg ml^−1^ HPV 16 DNA) tested in triplicate. Specimen RLU values were then converted into a ratio to the cutoff value. Specimens with a ratio <1.00 were considered negative. Specimens with ratios between 1.00 and 5.00 were systematically retested in duplicate and the mean of the three values was calculated. If discrepant RLU values were observed in the two tests, samples were tested in duplicate again (maximum three thawing cycles). Specimens with a final ratio ⩾1.00 were considered positive (HPV+). In the text, HPV always refers to the Hybrid Capture® II test. Therefore, an HPV− might refer to the absence of HPV virus or to an undetected type by the Hybrid Capture® II test or detection of the virus at an RLU ratio <1.00. All positive RLU values were collected and inserted into the database in order to compare the viral load expressed in RLU with the cytologic and biopsy results.

### Cytology

All the cytologic slides were read in the Cytopath Laboratory by one of 13 cytotechnicians who knew which cases in the study but did not have access to the HPV result (nor did any of the medical staff) before the cytologic report was sent to the gynaecologist. The cytologic interpretation was done according to the Bethesda 2001 recommendations (Solomon *et al*, 2002). Any result ⩾ASC-US was considered as a positive cytologic result (CYTO+). If either test was positive, a request was sent for colposcopy with biopsy. To permit computation of true test sensitivity and specificity, disease prevalence among the women with both tests negative was assessed by performing colposcopy and biopsy on 502 randomly selected members of this group. At the end of the study, all cytologic preparations associated with a subsequent HSIL biopsy result were reviewed. The number of positive spots was retrospectively determined on each slide. A positive spot was defined as a single cell or a group of cells with sufficient atypia to be reported as at least an AS-CUS.

### Biopsy

Before the beginning of the study, most of the 113 gynaecologists attended a formal review course on colposcopy procedure and interpretation taught by a Swiss expert colposcopist and educator. A biopsy was requested on all patients undergoing colposcopy: the biopsy would be directed if a lesion was noted (acetowhite epithelium, mosaicism, etc.), or random by strongly brushing the proximal endocervical canal if no lesion was visualised. In the latter case, the collecting device (a brush) was sent to the laboratory in formalin. At the laboratory, the brush was scraped with a scalpel and all material was paraffin blocked in order to obtain a histological preparation.

### Statistical methods

Percentage values were described with 95% asymmetrical binomial confidence intervals computed with the Clopper–Pearson exact method. Student's *t*-test was used to compare mean values with a threshold of 5% for *P*-value to be significant. In order to correctly estimate the sensitivity and specificity of both tests, a method for correcting biopsy verification bias was used. Verification bias is caused by the fact that the percentages of biopsy verification vary considerably among HPV/CYTO subsets because of practical limitations. Indeed, only 4% (502 out of 12 508) of women HPV−/CYTO− and 77% (1031 out of 1334) of women positive for at least one test, HPV+/CYTO−, HPV−/CYTO+ or HPV+/CYTO+, underwent colposcopy with biopsy. Given the low prevalence of disease in this population, the bias causes a falsely decreased specificity for both tests. The correction relies on the estimation of lesion frequency found in each subset HPV/CYTO if *all* patients had been colposcoped and biopsied. The adjusted value (AHCL) was derived from the formula *A*_HCL_=*F*_HCL_+*U*_HC_^*^(*F*_HCL_/(*F*_HC(Normal)_+*F*_HC(LSIL)_+*F*_HC(HSIL)_)) (Ratnam *et al*, 2000). L is the biopsy result. FHCL is the frequency of lesion L obtained from the study without adjustment within a given HPV/CYTO subset. *U*_HC_ is the number of unverified patients in the same subset either because the biopsy was not received or because the biopsy was unsatisfactory.

## RESULTS

### Cytology and HPV

**Figure 1:**
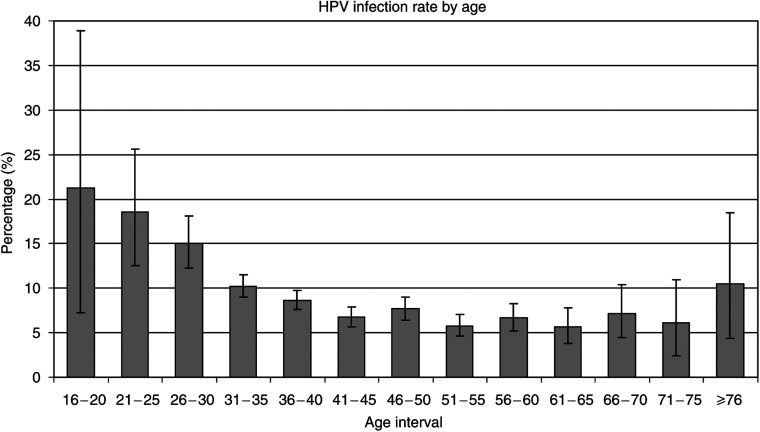
Percentage of HPV infection (*y*-axis) against age intervals (*x*-axis). 95% confidence intervals are superposed to grey bars. The highest HPV infection rates are found in the youngest women. The percentage progressively declines as age increases (*n*=13 840).

In total, 13 865 liquid cytology specimens were received in the laboratory. Of these, 13 842 were completed for both tests, CYTO and HPV. Overall, 9.6% of women (1334 out of 13 842) were positive for one or both tests (CYTO or HPV), 8.2% of women (1143 out of 13 842) were positive for HPV and 6.1% (841 out of 13 842) were positive for HPV alone (HPV+CYTO−). In all, 96.4% (13345 out of 13 842) of women were 30 years old or more, with a percentage of HPV infection of 7.9%. This percentage rose to 17.4% in the 495 women younger than 30 years. For two women, the age was not available. Mean age of the 13 840 women was 44.4 years (range 17–93). The higher frequency of HPV positivity in younger women is portrayed in Figure 1, where a progressive decline of HPV positivity is observed as age increases. All further results are unrelated to age. In total, 3.6% (493 out of 13 842) of women were positive for CYTO and 1.4% (191 out of 13 842) were positive for CYTO alone (HPV−CYTO+). Among the CYTO results, there were 243 ASC-US (1.8%), 14 atypical squamous cells: cannot exclude HSIL (ASC-H) (0.1%), 201 low-grade squamous intraepithelial lesion (LSIL) (1.5%), 30 high-grade squamous intraepithelial lesion (HSIL) (0.2%) and five atypical glandular cells (AGC) (0.04%).

### Biopsy results

Of the 1334 women with abnormal tests, 1086 (81.4%) underwent colposcopy with biopsy. Out of 1086, 1031 (95%) biopsies were satisfactory for evaluation. A total of 18.5% women (248/ out of 1334) refused to be colposcoped, missed appointments or underwent the follow-up in other laboratories. In order to verify the negative group (HPV−CYTO−), random invitations for colposcopy were regularly sent to women with both tests negative during the study. Since some women declined to participate, up to 700 invitations were sent before being able to gather the target number of 500. Finally, 502 women with normal tests were colposcoped and biopsied. These biopsy results were divided into three categories: normal, LSIL (CIN 1) and HSIL (CIN2/3). No invasive lesion was found on cytology and biopsy in any group. The details of the results are presented in Table 1, where biopsy results are crossed to CYTO and HPV results. Table 2 presents biopsy results according to the HPV/CYTO subsets. A key result is that 41.5% of all histologic HSIL lesions were found in the subset HPV+CYTO−.

### Sensitivity, specificity and negative predictive value of CYTO and HPV

The unbiased estimation of frequencies of lesions (normal, LSIL and HSIL) per HPV/CYTO subset for the entire study population (Table 3) permits calculation of unbiased sensitivity and specificity for detection of histologic HSIL (95% confidence interval in parentheses). Sensitivity is 58.7% (48.6–68.2) for CYTO and 97% (91.8–99.4) for HPV, while specificity is 96.9% (96.6–97.2) for CYTO and 92.4% (91.9–92.9) for HPV. The negative predictive value of CYTO and HPV are, respectively, 99.75 (99.67–99.83) and 99.98 (99.96–100). On the basis of the fact that no histologic HSIL lesion was found in the 502 randomly chosen women negative for both tests, the negative predictive value of the combination of negative tests HPV and CYTO estimated for all 13 842 patients is 100% (99.98–100).

### Viral load expressed as RLU and logarithm RLU, compared with cytological and biopsy results

**Figure 2:**
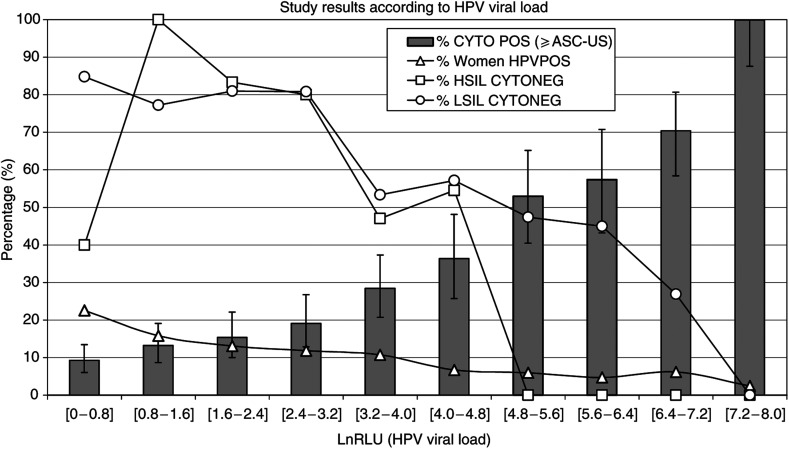
Percentage (*y*-axis) against HPV viral load of 1143 HPV+ samples (*x*-axis) reported as Logarithm value of Relative Light Unit (LnRLU). The ‘% women HPVPOS’ curve shows the distribution of the HPV+ samples among LnRLU intervals. In this study, most HPV+ samples have low LnRLU: near 50% of HPV+ samples are found in the first three LnRLU intervals. The grey bars represent the percentage per LnRLU interval of positive cytological preparation (⩾ASC-US); it shows a direct relationship between the HPV viral load and the probability for a Pap test to be positive. The HSIL CYTONEG and LSIL CYTONEG curves represent the percentage per LnRLU interval of false negative cytological preparations with subsequent biopsy proven lesion.

The 1143 HPV-positive results have RLU values ranging from 1 to 3180. The distribution of RLU values is nonlinear. Most RLU values are small: cumulatively, 22% of all the HPV-positive results are less than 2.2 RLU, 38% are less than 5.0 RLU and 51% are less than 11.2 RLU. In order to compare the HPV distribution values more easily against frequency, the RLU values were transformed using the natural logarithm function. Therefore, the RLU interval (1 : 3200) becomes the lnRLU interval (0 : 8). The latter was subdivided in 10 subintervals as illustrated in Figure 2. In this figure, the black line with small white triangles represents the percentage of HPV+ samples per lnRLU interval. This line shows that a majority of HPV-positive samples are associated with low HPV viral loads. The grey bars in Figure 2 represent the frequencies of positive CYTO against the HPV viral load expressed in lnRLU. A clearcut relationship is observed: the CYTO positivity frequency is directly related to the viral load. In the last subinterval [7.2–8.0], all the 28 HPV+ samples were always associated with a positive CYTO test (⩾ASC-US).

The black line with small white squares in Figure 2 represents the CYTO false negatives with subsequent biopsy proven HSIL. The 95% confidence intervals are not drawn (to avoid obscuring the chart) but are enumerated thereafter in square brackets. Per increasing lnRLU interval, the frequencies of histologic HSIL (CYTO negative) are 2/5=40% [12–77], 5/5=100% [56–100], 5/6=83% [35–99], 8/10=80% [44–97], 8/17=47% [23–72], 6/11=54% [23–83], 0/9=0% [0–28], 0/5=0% [0–45], 0/6=0% [0–39] and 0/5=0% [0–45]. For lnRLU values ⩾4.8, 25 histologic HSIL were found and all were detected by CYTO.

The black line with small white circles in Figure 2 represents the CYTO false negatives with subsequent biopsy proven LSIL. The 95% confidence intervals are not drawn as previously, but are enumerated thereafter in square brackets. Per increasing lnRLU interval, the frequencies of histologic LSIL (CYTO negative) are 67/79=84% [75–92], 44/57=77% [64–87], 34/42=80% [66–91], 38/47=80% [67–91], 16/30=53% [34–72], 16/28=57% [37–76], 9/19=47% [24–71], 9/20=45% [23–68], 7/26=27% [12–48] and 0/11=0% [0–24].

### Retrospective analysis of cytological preparations associated with HSIL

**Figure 3:**
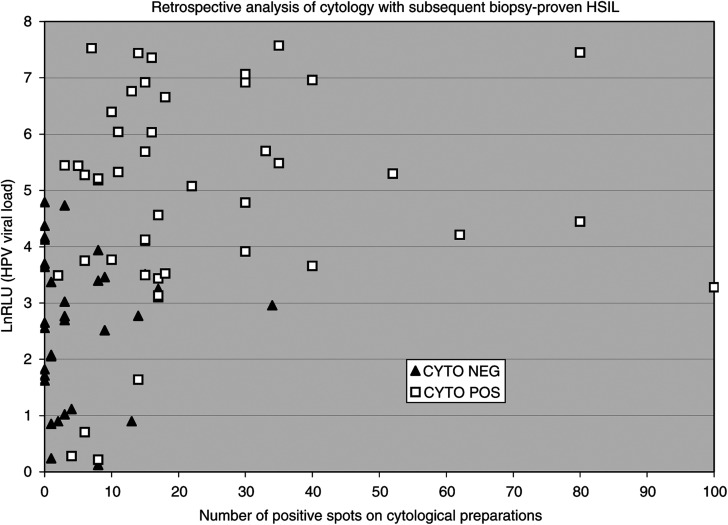
Retrospective analysis of all cytological preparations associated with subsequent histological HSIL. Logarithm values of RLU (*y*-axis) against number of positive cellular spots (*x*-axis) retrospectively counted on cytological preparations. False negative CYTO (black triangles) have a smaller viral load and fewer number of positive cellular spots than true positive CYTO (white squares). Both differences are statistically significant. However, a significant number of false negative cytological preparations (11 out of 34 (32%)) have no cellular spots despite a significant viral load (*n*=79).

Figure 3 portrays the retrospective analysis of the 79 cytological preparations (CYTO−HPV+ and CYTO+HPV+) associated with biopsy-proven HSIL (one cytological preparation, HPV+CYTO+, was not available for spot counting). The mean number of positive spots was 5.2 for HPV+/CYTO− cytological preparations (black triangles, *n*=34) and 23.1 for HPV+/CYTO+ cytological preparations (white squares, *n*=45). A total of 14% (11 out of 79) of cytological preparations HPV+/CYTO− did not show any positive spots. The mean lnRLU values were 2.6 for cytological preparations HPV+/CYTO− (black triangles, *n*=34) and 4.8 for HPV+/CYTO+ cytological preparations (white squares, *n*=45). Both differences (number of positive spots and lnRLU) were found significant with *t*-test (*P*<0.05). The two cytological preparations HPV−/CYTO+ associated to biopsy-proven HSIL had six and 10 positive spots (not shown in Figure 3).

## DISCUSSION

It is important to note that the methodology of the present study required that the collecting device be kept in the vial so that 100% of the cervical scrape was sent to the laboratory. The conventional smear, by selecting a random fraction of the cervical scrape, involves an important loss of material (Goodman and Hutchinson, 1996; Al-Awadhi *et al*, 2001). LBC technique may also reproduce the flaws of the conventional smear. It was demonstrated that when discarding the collecting device, a mean of 37% of the cervical scrape material is lost. Worse, often the lost material comes preferentially from the transformation zone (Bigras *et al*, 2003).

The relationship between the percentage of HPV infection and the age shown in Figure 1 is similar to those found in other studies except for the oldest group. This last interval is based on a reduced number of women as shown by its relatively wide confidence interval. Furthermore, it is hypothesised that this high frequency is probably biased because these older women are likely to be those who had previous abnormal tests and probably form a nonrandom sample of this age group.

### Contribution of HPV viral load study

At the sample level, the study of HPV viral load explains the reason that CYTO has inferior sensitivity. As shown in Figure 2, the probability of detecting abnormal cell(s) in an LBC preparation is a function of the associated HPV viral load. These results are concordant with the work of Schlecht *et al* (2003). As HPV is an intracellular virus, the viral load is proportional to the number of infected cells. Then, the higher the viral load, the greater the number of infected cells on the slide and the greater the probability for the LBC test to be positive. However, the viral load per cell varies between the LSIL and HSIL cells. LSIL is associated with active viral genome amplification with up to 1000 viral copies per keratinocyte (Garner-Hamrick and Fisher, 2002), while HSIL is associated with host DNA integration with few viral DNA per keratinocyte (Jeon *et al*, 1995). Then, an HSIL-associated sample would contain many more abnormal cells than an LSIL-associated sample with identical HPV viral load. This would explain why from 4.8 lnRLU value and higher (Figure 2), there are no more histologic HSIL false negative CYTO samples but still many LSIL false negative CYTO samples.

While some false negative cytological preparations can be explained by human misreading, most are not. False negative preparations would contain few abnormal cells and relatively low HPV viral load. Inspection of Figure 3 partly supports this hypothesis. First, it is reasonable, although arbitrary, to accept that missing up to three abnormal cells among 30 000–50 000 cells is independent of cytotechnician reading performance. Therefore, 64% (22 out of 34) of false negative CYTO with subsequent histologic HSIL were independent of cytotechnician reading performance. Furthermore, up to 14% (11 out of 79) of cytological preparations showed no abnormal cells despite relatively high viral loads. It is then hypothesised that infected cells are present on the slides but do not always show sufficient morphologic anomalies to be recognised. This concept is supported in the literature (Ward *et al*, 1990; Cramer *et al*, 1997; Salvia *et al*, 2004).

At the population level, the study of HPV viral load explains why cytology sensitivity varies considerably among reports. The relationship between the probability that a Pap test is abnormal and the viral load is thought to be independent from intrinsic factor related to this study. Conversely, the viral load distribution (Figure 2: black line with white triangles) is unique to the studied population. Its left-shifted pattern (indicating that a majority of HPV+ samples have small viral load) would be the hallmark of a low-risk population. If the same study was repeated with a high-risk population, it can be expected that the viral load distribution would be shifted to the right. Then, the number of abnormal cells per sample would be much more important and, consequently, the sensitivity gap between HPV and cytology for detection of HSIL would be much smaller. This idea is supported in the literature, as studies performed in a high-risk population demonstrated relatively small gaps (4, 10, 10 and 15%) (Belinson *et al*, 1999; Kuhn *et al*, 2000; Schiffman *et al*, 2000; Wright *et al*, 2000) compared with studies performed in a low-risk population with higher gaps (30, 38, 54 and 55%) (Ratnam *et al*, 2000; Clavel *et al*, 2001; Kulasingam *et al*, 2002; Petry *et al*, 2003).

### HPV/CYTO subsets

Derived from Table 2, the subsets HPV+/CYTO+ and HPV+/CYTO− represent, respectively, 2.18 and 6.07% of the studied population. HSIL form 19.2% of biopsies in the HPV+/CYTO+ subset and 5.1% in the HPV+/CYTO− subset. Combining these results, one computes a ratio women/HSIL of 5.2 for HPV+/CYTO+ and 19.6 for HPV+/CYTO− subsets. The large number of women without HSIL in the HPV+/CYTO− subset raises a difficulty in the clinical management. Wright *et al* (2004) have suggested an interim guidance for this issue. They suggested that colposcopy should not be performed in the setting routinely. HPV, along with cytology, should be repeated at 6–12 months. If either test is still positive, then colposcopy should be performed. Finding rare HSIL in the HPV−/CYTO+ subset illustrates that rare HPV subtypes not detected by HC2 may cause HSIL. Cytology must then still be part of screening strategy. No HSIL was found in 502 biopsies from the HPV−/CYTO− subset. This is concordant with the Guanacaste study (Bratti *et al*, 2004: 0 HSIL out of 150) and the Shanxi study (Belinson *et al*, 1999 (Dr R Gerald Pretorius, personal communication, January 2005): 0 HSIL out of 1274). In total, no HSIL was found in 1926 biopsies from HPV−/CYTO− subset. The 95% confidence interval, based on these 1926 biopsies, for the negative predictive value of the two tests together for histologic HSIL, is therefore 99.84–100.00%.

## CONCLUSIONS

This study found a higher sensitivity for HPV than liquid-based cytology testing in the detection of cervical cancer precursors. This adds to previous similar reports with the distinction that our study involved a nonacademic setting, thereby demonstrating that the method is robust and readily applicable as a routine test. By collecting all RLU values, a clearcut correlation between the probability for a Pap test to be positive and the associated HPV viral load was found. The findings can also explain how cytology sensitivity can vary among different studies since different populations will differ in their viral load distributions: at one extreme, cytology performs badly when the screened population is at low –risk, while at the other extreme, cytology performance would be much closer to that of HPV testing in a high-risk population. Rare HSIL are not detected by HC2, these cases being presumably related to HPV types that are not detected by the cocktail of probes used in HC2. The combination of Pap and HPV testing identifies all of the women harbouring HSIL that can be detected by the best colposcopic methods currently available, and thereby permits investigators and clinicians to focus their efforts on the population at risk, while reassuring the majority of women who are negative on both tests.

## Figures and Tables

**Table 1:** Biopsy results (*n*=1533) including control group crossed to Pap results (based on Bethesda 2001) and HPV results

	**PAP cytology results**						
**Biopsy**	**Normal**	**ASC-US**	**ASC-H**	**LSIL**	**HSIL**	**AGC**	**Total**
*HPV− (n=626)*							
AIS	0	0	0	0	0	0	0
CIN3	0	1	0	0	0	0	1
CIN2	0	1	0	0	0	0	1
CIN1	11	39	1	7	0	0	58
Normal	491	63	0	9	1	2	566

Total	502	104	1	16	1	2	

*HPV+ (n=907)*							
AIS*	0	1	0	0	0	2	3
CIN3	25	6	1	7	16	0	55
CIN2	9	2	2	3	6	0	22
CIN1	240	30	1	83	3	0	357
Normal	393	24	1	50	2	0	470

Total	667	63	5	143	27	2	

ASC-US=atypical squamous cells of undetermined significance; ASC-H=atypical squamous cells: cannot exclude HSIL; LSIL=low-grade squamous intraepithelial lesion; HSIL=high-grade squamous intraepithelial lesion; AGC=atypical glandular cells.

^*^The three AIS (*in situ* adenocarcinoma) were found combined with HSIL (CIN3) lesions within each biopsy.

**Table 2:** Biopsy results according to subsets HPV/CYTO

**Category results**	**Results (*n*)**	**Satisfactory biopsies (*n*)**	**Normal biopsies**	**LSIL biopsies**	**HSIL biopsies**
HPV−CYTO−	12 508	502	491 (12 233.9)	11 (274.1)	0 (0)
HPV−CYTO+	191	124	75 (115.5)	47 (72.4)	2 (3.1)
HPV+CYTO+	302	240	77 (96.9)	117 (147.2)	46 (57.9)
HPV+CYTO−	841	667	393 (495.5)	240 (302.6)	34 (42.9)

Total	13 842	1533	1036 (12 941.8)	415 (796.3)	82 (103.9)

Adjusted values (*A*_HCL_) for verification bias are in parentheses. They derived from the formula *A*_HCL_=*F*_HCL_+*U*_HC_^*^(*F*_HCL_/(*F*_HC(normal)_+*F*_HC(LSIL)_+*F*_HC(HSIL)_)). L is the biopsy result. *F*_HCL_ is the frequency of lesion L obtained from the study without adjustment within a given HPV/CYTO subset. *U*_HC_ is the number of patients in the same subset unverified either because the biopsy was not received or the biopsy was unsatisfactory. For instance, *A*_HPV+CYTO−(normal)_=*F*_HPV+CYTO−(normal)_+*U*_HPV+CYTO−_^*^(*F*_HPV+CYTO−(normal)_/(*F*_HPV+CYTO−(normal)_+*F*_HPV+CYTO−(LSIL)_+*F*_HPV+CYTO−(HSIL)_))=393+(841–667) ^*^ (393/(393+240+34))=495.5 (see text in statistical method for verification bias explanation).

HSIL=high-grade squamous intraepithelial lesion; LSIL=low-grade squamous intraepithelial lesion.

**Table 3:** Frequencies of true positive, true negative, false positive and false negative for HPV and CYTO tests (for HSIL detection) where HPV and CYTO represent adjusted values (*A*_HCL_, see Table 2 for biopsy verification bias)

**Test frequencies to detect CIN2/3 (HSIL) with related sensitivity and specificity adjusted with verification bias**				
**Result**	**HPV**	**CYTO**	**Sensitivity and specificity to detect HSIL**	
True positive	100.8	61.0	Sensitivity CYTO	58.7%
True negative	12695.9	13306.1	Specificity CYTO	96.9%
False positive	1042.2	432.0	Sensitivity HPV	97.0%
False negative	3.1	42.9	Specificity HPV	92.4%

95% confidence interval: sensitivity HPV=[91.8–99.4%], sensitivity CYTO=[48.6–68.2%], specificity HPV=[91.9–92.9%] and specificity CYTO=[96.6–97.2%].
